# Emergency cesarean delivery followed by extended left hepatectomy and right hepatic hemangioma enucleation for giant hepatic hemangioma with intratumoral hemorrhage in late pregnancy: a case report

**DOI:** 10.3389/fgwh.2026.1899670

**Published:** 2026-09-16

**Authors:** Yuting Xiao, Honghui Zhang, Weiming Yi, Chao Guo, Ting Dai, Chuang Peng, Xinli Zhang, Dan Liu

**Affiliations:** Department of Hepatobiliary Surgery, Hunan Provincial People’s Hospital, The First-Affiliated Hospital of Hunan Normal University, Hunan, China

**Keywords:** emergency management, hepatic hemangioma, late pregnancy, liver, tumor

## Abstract

Management of giant hepatic hemangioma (HH) complicated by intratumoral hemorrhage in late pregnancy is rarely reported and remains particularly challenging because maternal hemorrhagic compromise and fetal safety must be addressed simultaneously. We report the case of a 36-year-old gravida 2 para 1 woman at 33 weeks and 5 days of gestation who was admitted after a heterogeneous abdominal mass was detected on routine prenatal ultrasonography. Contrast-enhanced computed tomography (CT) demonstrated a giant lesion in the left hepatic lobe that was considered most consistent with HH complicated by intratumoral hemorrhage and an additional hemangioma in the right hepatic lobe, and laboratory testing revealed severe anemia and hypoalbuminemia. After blood transfusion, albumin supplementation, and multidisciplinary evaluation, the patient underwent emergency cesarean delivery followed by extended left hepatectomy and enucleation of the right hepatic hemangioma. Both the mother and the neonate recovered uneventfully, and no significant abnormalities were noted during 1 month of follow-up. This case highlights that in late pregnancy complicated by hemorrhagic giant HH, early recognition, rapid multidisciplinary decision-making, and timely combined obstetric and hepatic intervention may be lifesaving and can achieve favorable maternal and neonatal outcomes.

## Introduction

Hepatic hemangioma (HH) is one of the most common benign vascular lesions of the liver and is frequently detected incidentally on imaging. Most patients remain asymptomatic, and the lesion typically remains stable over time. Therefore, specific treatment is usually unnecessary. Only a minority of patients with giant HH develop symptoms related to mass effect, such as right upper abdominal discomfort, abdominal distension, or compression of adjacent organs. In some cases, further intervention may be required because of progressive enlargement, hemorrhage, or coagulation abnormalities (1).

Conservative follow-up remains the mainstay of management for HH. However, hemorrhagic events, particularly intratumoral hemorrhage or rupture-related bleeding, represent one of its rarest yet most serious complications. Once bleeding occurs, hemoglobin levels may decline rapidly, leading to hypovolemia, coagulopathy, and even hemorrhagic shock. In patients with rapid clinical deterioration or uncontrolled bleeding, the extent of the lesion and the severity of hemorrhage should be assessed promptly after initial resuscitation, and treatment options such as transcatheter embolization, surgical hemostasis, or hepatectomy should be selected according to the patient's overall condition (2).

Giant HH during pregnancy is itself uncommon, and reports of emergency management for intratumoral hemorrhage in late pregnancy are even more limited. Management in this setting is particularly challenging because it must address not only maternal blood loss, hypoperfusion, and hemodynamic instability, but also the risk of acute fetal hypoxia and adverse perinatal outcomes secondary to reduced placental perfusion. Therefore, early recognition of disease progression, prompt multidisciplinary coordination, and timely decisions regarding maternal safety, hemostatic intervention, and pregnancy termination are of considerable clinical importance. Here, we report the case of a 36-year-old woman at 33 weeks and 5 days' gestation who underwent emergency cesarean delivery followed by extended left hepatectomy for giant HH with intratumoral hemorrhage, together with enucleation of the right hepatic hemangioma, and ultimately achieved a favorable maternal and neonatal outcome. This case may help inform the management of similar patients.

## Case presentation

A 36-year-old gravida 2 para 1 woman at 33 weeks and 5 days of gestation was admitted after a heterogeneous abdominal mass had been detected on routine prenatal ultrasonography 6 days earlier. Her medical history was notable for a previous cesarean section and prior surgery for HH. After admission, contrast-enhanced CT revealed a giant heterogeneous mass in the left hepatic lobe measuring approximately 16 cm in diameter, which was considered most likely to represent HH complicated by intratumoral hemorrhage (Figure 1A). Contrast-enhanced CT also identified an additional hemangioma measuring approximately 10 cm in diameter in the right hepatic lobe.

**Figure 1:**
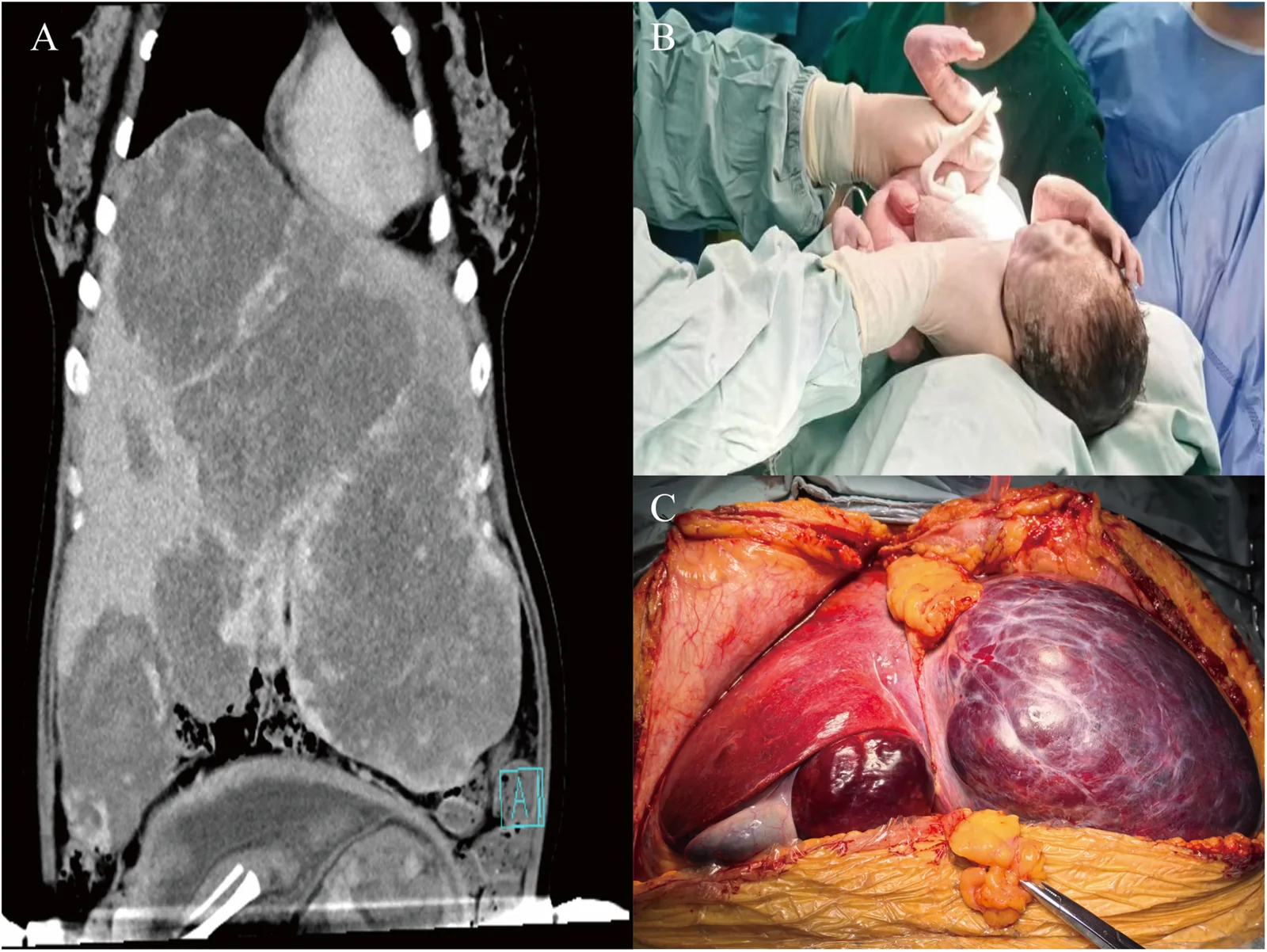
Imaging and intraoperative findings. **(A)** Contrast-enhanced CT demonstrating a giant hepatic hemangioma in the left lobe with intratumoral hemorrhage and an additional hemangioma in the right hepatic lobe. **(B)** Neonate delivered by emergency cesarean section. **(C)** Intraoperative view showing the giant hemangioma occupying the left hepatic lobe.

Laboratory investigations on admission showed marked anemia and hypoalbuminemia, with a hemoglobin level of 59 g/L and a serum albumin level of 24.55 g/L. Taken together, the imaging findings and laboratory abnormalities suggested an active hemorrhagic process with substantial maternal risk. Supportive treatment was initiated immediately, including transfusion of 5 units of leukoreduced packed red blood cells and administration of 10 g of albumin. Multidisciplinary consultation involving obstetrics, hepatobiliary surgery, anesthesiology, neonatology, and other relevant specialties focused on three key issues: maternal hemorrhagic risk, fetal viability at 33 weeks and 5 days of gestation, and the feasibility of definitive surgical control at our center. A consensus was reached to terminate the pregnancy and proceed directly to combined surgical management.

Emergency cesarean delivery was performed first, and a live infant weighing 1,810 g was delivered and transferred to the neonatology department for further care (Figure 1B). After closure of the uterine incision, the hepatobiliary surgery team performed extended left hepatectomy through a Mercedes incision (Figure 1C), together with enucleation of the right hepatic hemangioma. The total operative time was approximately 6 h 15 min. Intraoperative blood loss was approximately 4,000 mL, with 2,500 mL of autologous blood reinfused and 6 units of packed red blood cells transfused.

Postoperatively, the patient received comprehensive management, including antimicrobial therapy, fluid replacement, analgesia, and albumin supplementation. On postoperative day 2, repeat laboratory testing showed the following results: hemoglobin, 144 g/L; albumin, 26.3 g/L; alanine aminotransferase, 72.5 U/L; and aspartate aminotransferase, 158.2 U/L. No obvious coagulation abnormalities were observed. The postoperative course was uneventful, and the patient was discharged on postoperative day 29. At the 1-month follow-up visit, she reported no significant discomfort, and both the mother and infant had recovered well overall. Postoperative pathological examination confirmed cavernous hemangioma.

## Discussion

HH detected during pregnancy is usually found incidentally, and conservative follow-up remains appropriate for most patients. However, pregnancy creates a unique setting in which hormonal influences, increased circulating volume, and progressive uterine enlargement may alter the behavior of large lesions. Current literature suggests that asymptomatic lesions smaller than 10 cm can usually be monitored, whereas giant lesions or symptomatic lesions warrant closer surveillance and individualized planning (3, 4). Therefore, pregnancy itself does not mandate intervention, but it lowers the threshold for vigilance in patients with large or unstable HH.

Once hemorrhage occurs, HH becomes a time-sensitive maternal-fetal emergency. Intratumoral hemorrhage may represent an active bleeding state even in the absence of free rupture and can be accompanied by progressive anemia, pain, hypovolemia, and coagulation abnormalities (5, 6). In late pregnancy, maternal blood loss may also reduce placental perfusion and expose the fetus to secondary hypoxia or distress. Thus, in pregnant patients with giant HH, marked anemia plus imaging evidence of hemorrhage should prompt immediate reassessment rather than continued expectant management (4–6).

Published experience remains limited, and no standardized management algorithm has been established. Nevertheless, reported strategies generally fall into three patterns: damage-control surgery after emergency delivery with staged definitive hepatectomy, a mixed or stepwise approach combining initial operative control, embolization, and later resection, and immediate definitive surgery after delivery in selected patients with available expertise (6–9). In practice, management should be individualized and generally revolves around three key considerations: maternal hemodynamic stability, gestational age and fetal viability, and the availability of surgical and interventional expertise (4, 6–8).

In the present case, severe anemia (hemoglobin 59 g/L) and CT evidence of intratumoral hemorrhage suggested that the lesion was no longer static but had entered a potentially ongoing hemorrhagic phase. Because the gestational age had reached 33 weeks and 5 days, extrauterine survival of the fetus was reasonably achievable, and our center could provide coordinated obstetric, hepatobiliary, anesthetic, neonatal, and critical care support, emergency cesarean delivery followed immediately by extended left hepatectomy, with concomitant enucleation of the right hepatic hemangioma, was considered the most direct strategy to protect both mother and fetus while achieving definitive hemostasis.

Interventional embolization, particularly transcatheter arterial embolization (TAE), may serve as a bridge therapy, adjunctive measure, or alternative hemostatic option in selected cases (4, 7, 10, 11). However, its role in late pregnancy remains highly individualized because timing, fetal status, radiation concerns, the need for rapid hemostasis, and local resource availability all influence feasibility. The favorable maternal and neonatal outcomes in our case suggest that, in carefully selected patients managed at appropriately equipped centers, combined emergency delivery and definitive hepatic surgery can be a feasible option.

## Conclusion

Giant HH with hemorrhage in late pregnancy is extremely rare but potentially life-threatening. Early recognition, rapid multidisciplinary assessment, and individualized management are essential. In selected patients treated at appropriately equipped centers, emergency cesarean delivery followed by definitive hepatic surgery may represent a feasible and effective strategy to secure maternal hemostasis while optimizing fetal safety.

## Author's note

For transparency, the authors acknowledge that the clinical event described in this case report was previously reported by several local Chinese news media outlets as part of public news coverage of the patient's successful treatment. These reports were intended for general public communication and provided brief descriptions of the clinical event and surgical management. Relevant media reports include, but are not limited to, the following: Hunan Daily: https://m.voc.com.cn/xhn/news/202602/31628360.html; Xiaoxiang Morning News: https://www.xxcb.cn/m/video?id=csp6cdb712347c7620abea0b08317a3854a&sign=c03e9613a&t=1787657301; Sina: https://k.sina.cn/article_5563941882_14ba303fa02703qffy.html?wm=13500_0055&sinawapsharesource=newsapp&vt=4; Phoenix New Media: https://ishare.ifeng.com/c/s/v006GSDkrgTBZmEs25-_hoaUwOq7Fp77SsbWkYocY3PIKAHjgHF–woy–g3v6oKw-_GBEk0?spss=np&channelId=&aman=fgj043Qr3rFfgNdA04NYTAcwNjxk5ZHmNkuYTJghOTnJm1T00v. Time: https://moment.rednet.cn/pc/content/646046/60/15722756.html. Other local media outlets also reported or reproduced substantially similar information regarding this clinical event.

## Data Availability

The raw data supporting the conclusions of this article will be made available by the authors, without undue reservation.
